# Cellular mechanisms mediating activity‐dependent extracellular space shrinkage in the retina

**DOI:** 10.1002/glia.24228

**Published:** 2022-06-09

**Authors:** Pei‐Pei Chiang, Sidney P. Kuo, Eric A. Newman

**Affiliations:** ^1^ Department of Neuroscience University of Minnesota Minneapolis Minnesota USA

**Keywords:** AQP4 channels, extracellular space, glial cells, inwardly rectifying K^+^ channels, monocarboxylate transporter, Müller cells, Na^+^/HCO_3_^−^ cotransporter, Na^+^/K^+^/2Cl^−^ cotransporter, retina

## Abstract

Volume transmission plays an essential role in CNS function, with neurotransmitters released from synapses diffusing through the extracellular space (ECS) to distant sites. Changes in the ECS volume fraction (*α*) will influence the diffusion and the concentration of transmitters within the ECS. We have recently shown that neuronal activity evoked by physiological photic stimuli results in rapid decreases in ECS *α* as large as 10% in the retina. We now characterize the cellular mechanisms responsible for this ECS shrinkage. We find that block of inwardly rectifying K^+^ channels with Ba^2+^, inhibition of the Na^+^/K^+^/2Cl^−^ cotransporter with bumetanide, or block of AQP4 water channels with TGN‐020 do not diminish the light‐evoked ECS decrease. Inhibition of the Na^+^/HCO_3_^−^ cotransporter by removing HCO_3_^−^ from the superfusate, in contrast, reduces the light‐evoked ECS decrease by 95.6%. Inhibition of the monocarboxylate transporter with alpha‐cyano‐4‐hydroxycinnamate (4‐CIN) also reduces the ECS shrinkage, but only by 32.5%. We tested whether the swelling of Müller cells, the principal glial cells of the retina, is responsible for the light‐evoked ECS shrinkage. Light stimulation evoked a 6.3% increase in the volume of the fine processes of Müller cells. This volume increase was reduced by 97.1% when HCO_3_^−^ was removed from the superfusate. We conclude that a large fraction of the activity‐dependent decrease in ECS *α* is generated by the activation of the Na^+^/HCO_3_^−^ cotransporter in Müller cells. The monocarboxylate transporter may also contribute to the response.

## Main Points

- Light‐evoked shrinkage of retinal extracellular space is abolished by block of the Na^+^/HCO^3^ – cotransporter.
- Light stimulation evokes an increase in the volume of Müller glial cell processes which is also abolished by cotransporter block.

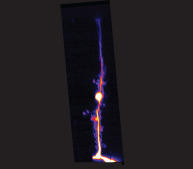

## 1 INTRODUCTION

Volume transmission, the diffusion of neurotransmitters through the extracellular space (ECS), plays an essential role in brain function. Neuromodulatory transmitters, including catecholamines, act in part by diffusing through the ECS after release from axonal varicosities (Sykova, 2004). In addition, spillover, a form of volume transmission where transmitters diffuse away from the synaptic cleft, results in the activation of extrasynaptic receptors and adjacent synapses (Sykova, 2004).

Volume transmission plays important roles in retinal function. During development, acetylcholine, released from starburst amacrine cells, and glutamate, released from bipolar cells, spread by volume transmission to generate spontaneous retinal waves (Blankenship et al., 2009; Blankenship & Feller, 2010; Feller et al., 1996). These waves of coordinated retinal ganglion cell (RGC) activity are essential for the formation of retinotopic maps in the brain (Torborg & Feller, 2005). In the adult retina, volume transmission of dopamine released from amacrine cells mediates circuit adaptation to light (Witkovsky, 2004) while activation of perisynaptic NMDA receptors by spillover contributes to RGC visual response properties (Chen & Diamond, 2002; Sagdullaev et al., 2006). Modulation of RGC excitability by release of ATP from Müller glial cells is also mediated by volume transmission (Newman, 2003). All of these processes are dependent on the properties of the ECS and, specifically, the ECS volume fraction (α), that fraction of the total volume of tissue occupied by the ECS (Nicholson & Hrabetova, 2017; Sykova & Nicholson, 2008). Reductions in ECS α will lead to higher neurotransmitter concentrations within the ECS.

We have recently demonstrated that neuronal activity evoked by physiological photic stimuli results in a rapid modulation of ECS α in the retina (Kuo et al., 2020). Light stimulation leads to a shrinkage of ECS α of up to 10% within tens of seconds and with latencies of ~1 s. Neuronal activity in the brain evoked by electrical stimulation (Prokopova‐Kubinova & Sykova, 2000; Ransom et al., 1985; Svoboda & Sykova, 1991), spreading depression (Mazel et al., 2002), and epileptiform activity (Colbourn et al., 2021; Slais et al., 2008) also result in decreases in ECS α.

Although the modulation of ECS α in the retina may influence important physiological processes, the mechanisms underlying this modulation are not understood. We now characterize the cellular mechanisms responsible for light‐evoked ECS α decreases in the retina. We find that the decrease is generated primarily by the action of the Na^+^/HCO_3_^−^ cotransporter. Our results suggest that activation of the cotransporter causes the processes of Müller glial cells to swell, leading to a reduction in ECS α.

## 2 METHODS

### 2.1 Ethics statement

All experimental procedures were approved by and adhered to the guidelines of the Institutional Animal Care and Use Committee of the University of Minnesota.

### 2.2 Retinal preparation

All experiments were performed on tissue from 8 to 12 week old male and female C57BL/6 mice. Detailed methods were described previously (Kuo et al., 2020). Briefly, eyes were enucleated and hemisected. The ventral portion of eyecups were trimmed down to a ~3 × 3 mm square and mounted flat onto a poly‐L‐lysine coated coverslip on the bottom of a perfusion chamber, vitreal surface up. The tissue was secured in place with a harp and was superfused at ~7 ml/min with warmed (~32°C), oxygenated aCSF.

### 2.3 Solutions and drugs

The bicarbonate‐buffered aCSF contained (in mM): 125 NaCl, 2.5 KCl, 1.2 CaCl_2_, 1.5 MgCl_2_, 1.25 NaH_2_PO_4_, 20 glucose, 0.5 l‐glutamine, 0.4 sodium ascorbate, 26 NaHCO_3_, bubbled with 95% O_2_/5% CO_2_ (pH 7.35; 300–310 mOsm). For HEPES‐buffered aCSF, the NaHCO_3_^−^.was replaced with 10 mM HEPES and NaCl increased to 151 mM, bubbled with 100% O_2_ (pH 7.35). For bicarbonate and HEPES‐buffered aCSF, 10 mM HEPES was added to the bicarbonate aCSF and the NaCl concentration decreased to 117 mM. The membrane‐impermeant fluorescent dye calcein (0.1 mM; Sigma #C0875) was added to the aCSF when measuring ECS α. The patch pipette solution for patching onto Müller cells contained (in mM): 120 K‐gluconate, 4.5 MgCl_2_, 9 HEPES, 0.1 EGTA, 14 tris_2_‐phosphocreatine, 4 Na_2_‐ATP, 0.3 tris‐GTP, sucrose to bring the solution to 285 mOsm, 20 μM Alexa Fluor 594 Hydrazide (Invitrogen #A14038) (pH 7.25).

Pharmacological agents added to the aCSF were used to block ion channels and transporters. Inwardly rectifying K^+^ channels were blocked with 200 μM BaCl_2_; the Na^+^/K^+^/2Cl^−^ cotransporter with 30 μM bumetanide (Tocris #3108), the Na^+^/HCO_3_^−^ cotransporter with 300 μM DIDS (4,4′‐diisothiocyanatostilbene‐2,2′‐disulfonate, Sigma #D3514), 50 μM S0859 (Sigma #SML0638) and 200 μM harmaline (Sigma #51330) or by replacing the HCO_3_^−^‐buffered aCSF with the HEPES‐buffered aCSF; the monocarboxylate transporter with 500 μM 4‐CIN (alpha‐cyano‐4‐hydroxycinnamate, Sigma #C2020); aquaporin‐4 (AQP4) water channels with 20 μM TGN‐020 (Sigma #SML0136).

### 2.4 Two‐photon imaging and visual stimulation

A custom‐built two‐photon laser scanning (2P) microscope equipped with a 25X, 1.05 NA objective (XLMPLN25XWLP2; Olympus) was used to image calcein in the ECS and Müller cell morphology. Two‐photon excitation of calcein was achieved using a Ti:sapphire laser (Chameleon Vision, Coherent) tuned to 800 nm (2–6 mW post‐objective laser power) with calcein fluorescence emission detected using a 500–550 nm bandpass filter. The z‐axis resolution of the microscope, measured by imaging 0.175 μm diameter fluorescent beads, was 1.4 μm (full‐width at half‐maximum of the bead fluorescence profile; Kuo et al., 2020). Alexa Fluor 594‐filled Müller cells were imaged with 810 nm excitation (1.2 mW post‐objective laser power) and a 570–640 nm bandpass emission filter. Images (512 × 512 pixels) were acquired at a 5 Hz frame rate.

When measuring light‐evoked ECS α changes and Müller cell process swelling, retinas were stimulated through the microscope objective with a 60 s, 5 Hz (50% duty cycle), 405 nm flickering light from a laser diode (L405P150; ThorLabs) projected through a diffuser and circular mask to produce a 100 μm diameter spot on the retina. Stimuli produced a photon flux of 15.3 × 10^5^ photons/(μm^2^ × s). Assuming a cone collecting area of 1.0 μm^2^ (Naarendorp et al., 2010), this was equivalent to 1.8 × 10^5^ cone opsin isomerizations per short wavelength‐sensitive cone per second (P*/[S cone × s]).

### 2.5 Measurement of ECS *α*

Eyecup pieces were superfused with aCSF containing the membrane‐impermeant fluorescent dye calcein (0.1 mM). ECS α was determined by measuring the spatially integrated calcein fluorescence intensity within an optical section of the retina and comparing it to the fluorescence of the aCSF above the retina, as described previously (Kuo et al., 2020). The dye equilibrates within the ECS of the retina within 5 min. Assuming that the dye fills all of the ECS and that the dye concentration within the ECS equals the concentration in the aCSF, then

$$\alpha =F_{\text{Retina}}/F_{\text{Perfusate}}$$

where *F* is the mean calcein fluorescence signal in an imaged plane. Calcein fluorescence measurements were corrected for depth‐dependent signal attenuation, as described previously (Kuo et al., 2020). Light‐evoked changes in ECS α were measured from the peak of the change to the pre‐stimulus baseline. All measurements were made in the middle lamina of the inner plexiform layer (IPL), approximately 25 μm beneath the vitreal surface of the retina.

### 2.6 Measurement of Müller cell process swelling

Stimulus‐evoked changes in the volume of Müller cell processes were monitored by measuring the integrated brightness of dye‐filled Müller cells. If the concentration of the fluorescent dye within the cell processes remains constant, the volume of the processes will be proportional to the total fluorescence brightness (Henneberger et al., 2020; Medvedev et al., 2014).

Müller cells were labeled intracellularly with Alexa Fluor 594 via dialysis through whole‐cell patch pipettes (5–8 MΩ impedance) attached to the endfeet of individual Müller cells at the vitreal surface of the retina. The membrane patch was ruptured and the cell held in current clamp mode as the Alexa dye filled the cell. Cell volume measurements were made 15–20 min after achieving whole‐cell mode to ensure complete dye‐filling. Müller cell processes were imaged in the middle of the IPL and did not include cell somata, which are in the inner nuclear layer. Fluorescence brightness was measured in a single focal plane in a region of interest that encompassed the fine processes radiating from the central stalk of the Müller cell but excluded the stalk itself (e.g., Figure 4b).

### 2.7 Measurement and analysis of retinal ganglion cell light responses

Retinal ganglion cell spiking activity was measured using loose cell‐attached extracellular patch recordings with pipettes (8–10 MΩ) filled with bicarbonate‐buffered aCSF. Recordings were acquired using a Multiclamp 700A amplifier (Molecular Devices) and Symphony Data Acquisition software (https://symphony-das.github.io). ON‐sustained RGCs were targeted for recordings by their large soma diameter (~15–20 μm) and characteristic sustained spiking responses to light step increments (Murphy & Rieke, 2006; Van Wyk et al., 2009). Visual stimuli were presented by projecting the output of a DLP projector illuminated by a 405 nm peak wavelength LED (PRO4500, Wintech Digital) through the microscope objective onto the retina. Visual stimuli were generated using Stage Visual Stimulation System software (https://stage-vss.github.io) and consisted of 300 μm diameter circular spots centered on the RGC soma that were presented for 0.5 s with 2 s between trials on a constant background light intensity that caused ~2400 P*/(S cone × sec)). Spike counts for each contrast were averaged over 10 trials. Stimulus contrast, *c*, was defined as:

$$c=\left(\frac{I-b}{b}\right)\times 100\%$$

where *I* is intensity of the spot stimulus and *b* is background light intensity. For each recorded cell, the number of spikes that occurred during spot presentations at different stimulus contrasts and in control versus drug conditions were normalized to the maximal spike count for a 400% contrast stimulus under control conditions. RGC contrast sensitivity was quantified by fitting mean normalized spike responses, *R*, at each stimulus contrast, *c*, with the Hill equation:

$$R\left(c\right)=R_{\max}\times \left(\frac{1}{1+{\left(\frac{c_{\text{half}}}{c}\right)}^{n}}\right)$$

where *R*_max_ is the maximal normalized spiking response, *c*_half_ is the contrast at half‐saturation of the response, and *n* defines the slope of the contrast‐response curve.

### 2.8 Experimental design and statistical analysis

Sample size, *n*, in all cases except RGC spike recordings, represents trials from individual eyecups, each from a separate mouse. In experiments measuring RGC light responses, *n* represents the number of RGCs that were recorded for each experimental group. Significance between control, experimental, and wash conditions was determined using paired *t*‐tests. Differences were considered significant at *p* < 0.05. Summary data are shown as individual trials and mean ± SEM. Statistics were performed using MATLAB (2017b). Results from male and female mice were pooled as there were no statistically significant differences between the two sexes.

## 3 RESULTS

We previously demonstrated that a flickering light stimulus evokes a rapid decrease in ECS α in the mouse retina (Kuo et al., 2020). We have now explored the cellular mechanisms leading to this ECS α decrease. Within the brain, changes in ECS α evoked by neuronal activity are believed to be generated largely by changes in the volume of astrocytes (MacAulay, 2020, 2021). Various mechanisms have been proposed to account for activity‐dependent volume changes in astrocytes in the brain, including the flow of water through AQP4 channels (Nagelhus et al., 2004), or through the action of the Na^+^/HCO_3_^−^ cotransporter or the monocarboxylate transporter (Larsen & MacAulay, 2017; MacAulay, 2020). Here, we investigate the mechanisms by which light‐evoked changes in ECS α are generated in the retina and whether the changes are due to the swelling of Müller cells, the principal glial cells of the retina (Newman & Reichenbach, 1996).

**FIGURE 1:**
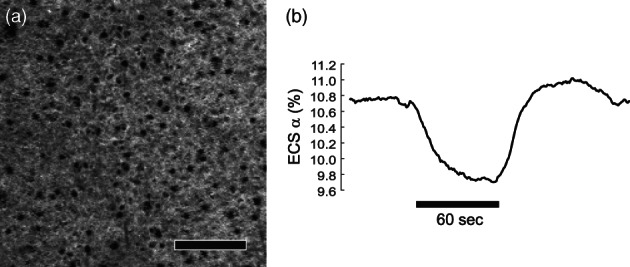
Measurement of the light‐evoked ECS α decrease. (a) Two‐photon microscopy image of the middle lamina of the inner plexiform layer, labeled with the fluorescent dye calcein. Calcein is restricted to and labels the ECS. The small black circles in the image are unlabeled cross sections of Müller cell stalks. Scale bar, 20 μm. (b) Time course of the light‐evoked ECS α decrease. ECS α values are calculated from the integrated calcein brightness in the image divided by the fluorescence brightness of the aCSF (Kuo et al., 2020). The horizontal bar indicates the time course of the light stimulus.

Retinas were superfused with an aCSF containing the membrane‐impermeant fluorescent dye calcein, which rapidly equilibrates with the ECS in the retina. Changes in ECS α were monitored by measuring the total integrated fluorescence of the dye in an optical section of the retina (Figure 1a). ECS α changes were monitored in the middle lamina of the IPL, where large light‐evoked α decreases are observed (Kuo et al., 2020). We used a standard light stimulus, a 100 μm diameter, 5 Hz flickering light of 1.8 × 10^5^ P*/(S cone × s) intensity and 1 min duration, which reliably produced a large, transient decrease in ECS α (Figure 1b). Light stimulation evoked an 8.7% reduction in ECS α, decreasing from 10.48 ± 0.11% to 9.57 ± 0.10% (*n* = 22), similar to what has been observed previously (Kuo et al., 2020).

### 3.1 Ion channel and transporter mechanisms

#### 3.1.1 Inwardly rectifying K^+^ channels

**FIGURE 2:**
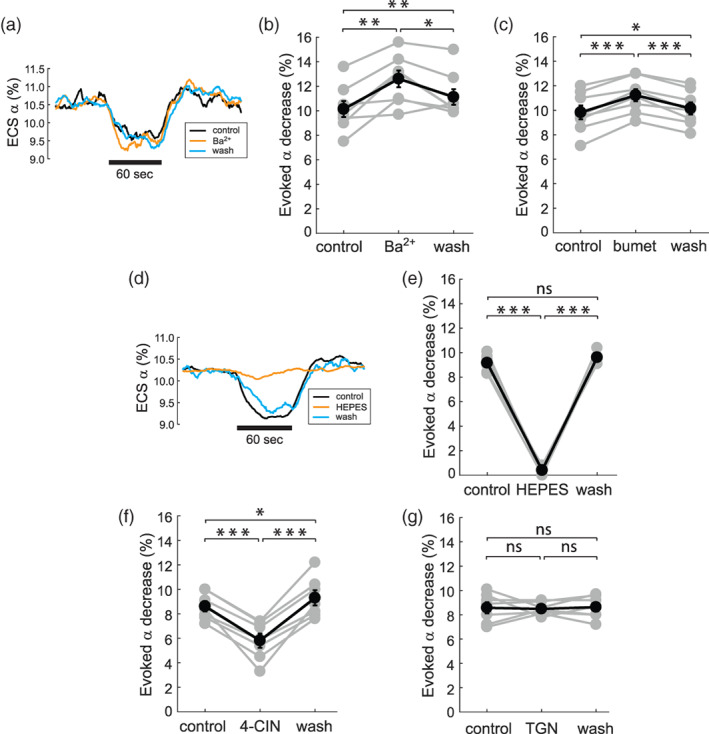
Mechanisms mediating light‐evoked extracellular space shrinkage. Example traces (a) and summary (b) showing that the ECS α decrease evoked by light stimulation is enhanced by the Kir channel blocker Ba^2+^ (200 μM); *n* = 8. The effect is reversible. (c) The Na^+^/K^+^/2Cl^−^ cotransporter blocker bumetanide (30 μM) also enhances the *α* decrease and is reversible; *n* = 8. Example traces (d) and summary (e) showing that the light‐evoked ECS α decrease is abolished when the Na^+^/HCO_3_^−^ cotransporter is blocked by substitution of a HEPES‐buffered aCSF for the HCO_3_^−^ buffered (control) aCSF; *n* = 6. The effect is reversible. (f) The monocarboxylate transporter blocker 4‐CIN (500 μM) reduces but does not eliminate the α decrease; *n* = 7. The effect is reversible. (g) the AQP4 blocker TGN‐020 (20 μM) has no effect on the α decrease; *n* = 7. In example traces (a and d) the experimental and wash traces have been offset vertically so that their baselines coincide with the control trace. Horizontal bars indicate the time course of the light stimulus. In summary plots, the change in ECS α in individual trials (gray) and the mean ± SEM values (black) are shown. *p ≤ .05; **p ≤ .01; ***, *p* ≤ .001; ns, not significant

We first explored whether light‐evoked ECS α decreases were reduced by blocking inwardly rectifying K^+^ (Kir) channels, which are expressed primarily in Müller cells in the retina (Ishii et al., 1997; Kofuji et al., 2000). Kir channels were blocked with 200 μM Ba^2+^, which effectively blocks Müller cell Kir channels (Newman, 1989). Addition of Ba^2+^ to the aCSF produced a 7.2 ± 2.8% decrease in the basal, steady state ECS α, suggesting that Kir channels contribute to volume regulation of glial cells or neurons in the retina. However, Ba^2+^ did not reduce the light‐evoked α decrease. Rather, the light‐evoked ECS α decrease was enhanced by 24.3% in the presence of Ba^2+^, with the α decrease growing from 10.14 ± 0.65% in control conditions to 12.61 ± 0.69% in Ba^2+^ (Figure 2a,b). The effect of Ba^2+^ was reversible, with the ECS α decrease returning toward the control value when Ba^2+^ was removed.

#### 3.1.2 Na^+^/K^+^/2Cl^−^ cotransporter

We next examined the role of the Na^+^/K^+^/2Cl^−^ cotransporter in generating the light‐evoked ECS α decrease. The NKCC1 transporter is expressed in Müller cells in early development but largely in neurons in the adult retina (Vardi et al., 2000; Zhang et al., 2007). We used bath application of bumetanide (30 μM) to block the transporter. Bumetanide did not alter the steady state ECS α. Bumetanide was also ineffective in reducing the light‐evoked ECS α decrease. In the presence of bumetanide, the light‐evoked α decrease was increased by 14.2%, from 9.83 ± 0.57% to 11.23 ± 0.50% (Figure 2c). The effect of bumetanide was also reversible, with the ECS α decrease returning toward the control value when bumetanide was removed.

#### 3.1.3 Na^+^/HCO_3_^−^ cotransporter

**FIGURE 3:**
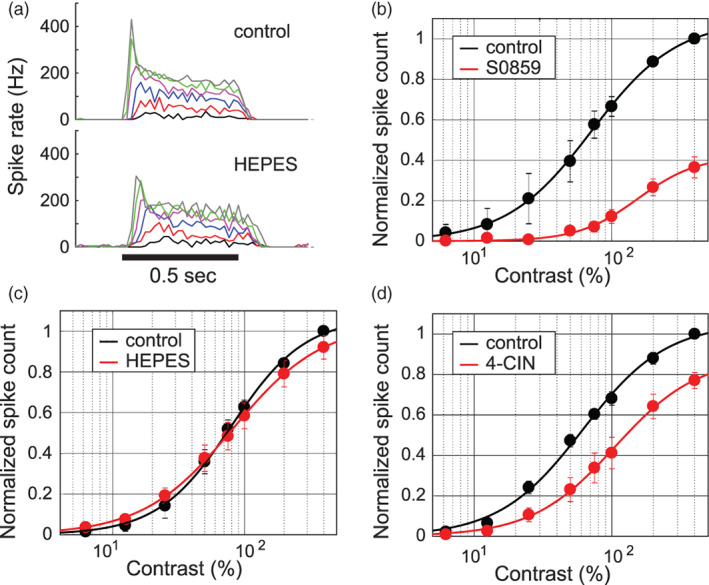
Changes to light‐evoked neuronal activity induced by pharmochological agents. Spike responses of ON‐sustained RGCs to a 0.5 s, 300 μm diameter spot flash of varying contrasts are shown. (a) Peri‐stimulus time histograms of spike responses of an example ON‐sustained RGC in control (top panel) and HCO_3_^−^ ‐free (HEPES‐buffered; bottom panel) solutions. Colored traces show spiking responses to stimuli of increasing contrast, from 12.5% to 400%. The horizontal bar indicates timing of the spot presentation. (b–d) Summary contrast sensitivity plots of RGC spike responses in control and test solutions from recordings as in (a). Normalized spike counts are shown as mean ± SEM (symbols) and are fit by the Hill equation (solid lines; see methods for details). (b) RGC responses in control and S0859 aCSF; *n* = 3 cells. (c) RGC responses in control (HCO_3_^−^) and HEPES‐buffered aCSF; *n* = 6 cells. (d) RGC responses in control and 4‐CIN aCSF; *n* = 6 cells

We examined the role of the Na^+^/HCO_3_^−^ cotransporter, which is highly expressed in Müller cells and regulates both Müller cell intracellular pH as well as extracellular pH in the retina (Newman, 1996, 1999). We initially evaluated the role of the Na^+^/HCO_3_^−^ cotransporter by inhibiting it with DIDS (300 μM). The drug reduced the light‐evoked ECS α decrease to near zero. This action could be due to a direct block of Na^+^/HCO_3_^−^ cotransport in Müller cells (Newman, 1991). However, DIDS is non‐selective and its effect on ECS α could be due instead to its action on retinal neurons. Indeed, DIDS has been shown to reduce photoreceptor and bipolar cell light responses in amphibians and fish (Jones & Palmer, 2011; Koskelainen et al., 1993). We therefore examined whether bath application of DIDS alters visual sensitivity of the retina by recording the light‐evoked spiking activity of ON‐sustained RGCs, which reflects the integrated light‐evoked activity of all upstream retinal neurons (photoreceptors, horizontal cells, bipolar cells, amacrine cells) as well as the intrinsic properties of the recorded RGC (Figure 3a, top panel). We found that DIDS completely abolished light‐driven spiking in ON‐sustained RGCs (*n* = 3; not shown). Thus, its effect on light‐evoked ECS α responses may be due to disruption of light‐driven neuronal activity.

We also examined the effects of S0859, a more selective Na^+^/HCO_3_^−^ cotransporter inhibitor than DIDS (Ch'En et al., 2008). In the presence of S0859 (50 μM), the light‐evoked α decrease was reduced by 85%. However, S0859 also strongly affected light‐evoked ON‐sustained RGC spiking. In the presence of S0859, the maximal light step‐evoked spike response (*R*_max_) was reduced by 58% and contrast‐sensitivity was reduced, with the contrast that produced a half‐maximal spike response (*c*_half_) shifting from 71.8% to 155.3% contrast (Figure 3b). We tested whether the effect of S0859 on RGC activity was due to disruption of pH regulation in the retina. Retinas were superfused with aCSF buffered with both HCO_3_^−^ and HEPES in order to maintain normal pH in the presence of Na^+^/HCO_3_^−^ cotransporter block by S0859. However, RGC activity was reduced by a similar extent in the presence of S0859 (*n* = 2; not shown). Therefore, as with DIDS, we cannot rule out that the reduction in light‐evoked ECS α was not simply a result of a drug‐induced decrease in retinal light sensitivity. We also attempted to test the role of the Na^+^/HCO_3_^−^ cotransporter with harmaline, which is another antagonist of the cotransporter (Grassl et al., 1987). However, harmaline (200 μM) in solution with calcein formed a fluorescent precipitate within the retina and thus could not be used to measure light‐evoked ECS α changes with our technique.

One manipulation was successful in examining the role of the Na^+^/HCO_3_^−^ cotransporter in generating the ECS α decrease. Transporter activity was blocked by removing HCO_3_^−^ from the aCSF; the HCO_3_^−^ ‐buffered aCSF was replaced with a HEPES‐buffered aCSF. This manipulation blocks the activity of the Na^+^/HCO_3_^−^ cotransporter in Müller cells (Newman, 1991). Blocking the cotransporter by removing HCO_3_^−^ raised the steady state ECS α value by 24.2 ± 14.2%. Removing HCO_3_^−^ effectively reduced the light‐evoked α decrease. When superfused with the HEPES‐buffered aCSF, the evoked α decrease was reduced by 95.6%, from 9.17 ± 0.30% to 0.40 ± 0.14% (Figure 2d,e). The block was completely reversible. When the HEPES‐buffered aCSF was once again replaced with the HCO_3_^−^ ‐buffered aCSF, the evoked α decrease recovered to 104.6% of the control value.

Importantly, removal of HCO_3_^−^ from the aCSF had little effect on neuronal responses. When the HCO_3_^−^ ‐buffered aCSF was replaced with a HEPES‐buffered aCSF, maximal spike responses (*R*_max_ = 107% in control aCSF vs 104% in HEPES‐buffered aCSF) and contrast sensitivity (*c*_half_ = 79.6% vs. 81.7% in control vs. HEPES‐buffered aCSF, respectively) were nearly identical to responses in HCO_3_^−^ ‐buffered aCSF (Figure 3a,c). These results rule out the possibility that the strong reduction of the light‐evoked ECS α decrease when HCO_3_^−^ was removed from the bath solution (Figure 2d,e) could be due to indirect effects on the visual response properties of retinal neurons.

#### 3.1.4 Monocarboxylate transporter

We evaluated the role of the monocarboxylate transporter (MCT) in generating the evoked ECS α decrease. MCT1 and MCT2 are expressed in Müller cells and photoreceptors in the retina (Bergersen et al., 1999; Gerhart et al., 1999). The monocarboxylate transporter was blocked with 4‐CIN (500 μM). In the presence of 4‐CIN, the steady state ECS α was unchanged. 4‐CIN reduced the evoked α decrease by 32.5%, from 8.61 ± 0.42% to 5.81 ± 0.58% (Figure 2f). This effect was reversible, with the evoked α decrease returning to 107.5% of the control value following washout of 4‐CIN.

We tested whether addition of 4‐CIN to the aCSF reduced light‐evoked neuronal activity. 4‐CIN reduced the maximal spike response by 16%, and reduced the contrast sensitivity (Figure 3d; *c*_half_ shifted from 61.6% to 105.6% contrast).

#### 3.1.5 Aquaporin‐4

We tested whether AQP4 water channels, which are expressed in astrocytes and Müller cells (Nagelhus et al., 1998; Nielsen et al., 1997) contribute to the generation of light‐evoked α variations in the retina. AQP4 channels were blocked with TGN‐020 (20 μM), a potent AQP4 inhibitor (Huber et al., 2009). In the presence of TGN‐020, the steady state ECS α was unchanged. The light‐evoked α decrease was unchanged as well, equaling 8.57 ± 0.45% in control aCSF and 8.49 ± 0.20% in TGN‐020 (Figure 2g).

### 3.2 Müller cell swelling

**FIGURE 4:**
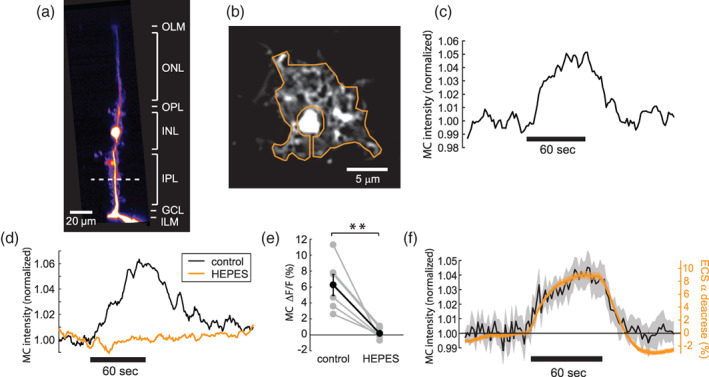
Müller cell swelling contributes to light‐evoked extracellular space shrinkage. (a) Vertical reconstruction of a z‐stack of 2P images of a Müller cell filled with Alexa Fluor 594. The tip of the patch pipette, attached to the Müller cell endfoot, is visible at the bottom. The dashed line shows the approximate location in the middle of the IPL where ECS α changes were measured and Müller cell processes were imaged. ILM, inner limiting membrane; GCL, ganglion cell layer; IPL inner plexiform layer; INL, inner nuclear layer; OPL outer plexiform layer; ONL, outer nuclear layer; OLM, outer limiting membrane. (b) Example 2P image of an Alexa Fluor 594‐labeled Müller cell from the middle of the IPL. The orange line outlines the region of interest (ROI) used to measure the fluorescence intensity of Müller cell processes. The central stalk of the Müller cell is excluded from the ROI. The contrast of the image has been increased for illustrative purposes. (c) Example trace of the time course of Müller cell process intensity in response to a photic stimulus. Light stimulation evoked a 5% increase in the fluorescence of the processes, indicating Müller cell swelling. The horizontal bars in (c), (d) and (f) indicate the time course of the light stimulus. (d) Example traces from a Müller cell showing the light‐evoked increase in Müller cell process intensity. The increase in intensity in control solution (black line) is abolished when the Na^+^/HCO_3_^−^ cotransporter is blocked by substitution of a HEPES‐buffered aCSF for the HCO_3_^−^ buffered (control) aCSF (orange line). (e) Summary showing the effect of HEPES substitution on the light‐evoked increase in Müller cell process intensity. The increase in intensity is abolished. Individual trials (gray) and the mean ± SEM values (black) are shown; *n* = 6. **, *p* ≤ .01. (f) the time course of the light‐evoked increase in Müller cell process intensity (black, mean ± SEM; eight cells) compared to that of the light‐evoked decrease in ECS α (orange, mean ± SEM; seven retinas). The ECS α trace has been inverted and the vertical scale adjusted to match the Müller cell trace. The two responses closely follow each other, except for the post‐stimulus overshoot of the ECS α decrease, seen as a negative dip in the orange trace.

In the brain, the swelling of astrocytes is largely responsible for generating ECS α decreases evoked by neuronal activity (MacAulay, 2020). In the retina, astrocytes are largely restricted to the nerve fiber layer at the inner border of the retina. In their place, Müller cells, which span the entire retinal depth (Figure 4a), assume many of the functions of astrocytes (Newman & Reichenbach, 1996). Müller cells are a likely candidate for mediating the light‐evoked ECS α decrease as they express both the Na^+^/HCO_3_^−^ transporter (Newman, 1996, 1999) and the monocarboxylate transporter (Bergersen et al., 1999; Gerhart et al., 1999).

We evaluated the role of Müller cells in generating the evoked α decrease by examining changes in their volume. We patched onto Müller cells and filled them with a fluorescent dye in order to visualize their processes (Figure 4b). Patched Müller cells were identified by a number of unique characteristics. (i) They had a highly negative resting membrane potential averaging −86.6 ± 0.7 mV (*n* = 13), (ii) they generated prominent slow depolarizations of 3.4 ± 0.2 mV (*n* = 5) at both the ON and OFF of light, (iii) they had a unique morphology, including a stalk which extended from the inner limiting membrane to the outer limiting membrane, a polygonally‐shaped soma in the middle of the inner nuclear layer, fine processes extending laterally from the stalk in the IPL, and microvilli extending from the outer limiting membrane into the subretinal space (Figure 4a,b).

If Müller cells generate the light‐evoked ECS α decrease, light stimulation should cause their processes to swell. We examined the fine processes of Müller cells in the middle sublamina of the IPL, where ECS α decreases were measured. We monitored the volume of these processes by measuring the integrated brightness of the dye‐filled processes in regions of interest that encompassed the processes but excluded the large stalk (Figure 4b). This technique has been used previously to monitor structural remodeling of astrocyte processes (Henneberger et al., 2020; Medvedev et al., 2014).

We found that light stimulation evoked increases in the brightness of Müller cell processes, indicating an increase in the volume of the processes (Figure 4c). The mean increase in brightness was 6.3 ± 1.3% (*n* = 6). If this increase in the volume of the Müller cell fine processes is responsible for generating the evoked ECS α decrease, then manipulations which block the α decrease should also block the increase in Müller cell process volume. This proved to be the case. Substituting a HEPES‐buffered aCSF for a HCO_3_^−^ ‐buffered aCSF, which almost completely eliminates the light‐evoked α decrease, abolished the increase in Müller cell processes brightness, with the brightness increase being reduced by 97.1% to 0.18 ± 0.28% (Figure 4d,e).

If the increase in Müller cell process volume generates the evoked α decrease, then the time course of the two responses should be similar. Again, this proved to be true. The time course of the volume increase in Müller cell processes closely followed the decrease in ECS α. The two had similar onset latencies, rise times, and decay times (Figure 4f). As described previously (Kuo et al., 2020), the light‐evoked decrease in ECS α is followed by a rebound overshoot, where ECS α briefly increases above baseline when the stimulus is turned off (Figure 4f, negative dip in orange trace). The evoked change in the volume of Müller cell processes does not display this overshoot (Figure 4f, black trace). Thus, it is likely that this rebound component of the ECS α change is generated by a non‐Müller cell mechanism.

## 4 DISCUSSION

We have investigated the cellular mechanisms responsible for generating the light‐evoked decrease in ECS α in the retina. We found that influx of K^+^ into retinal cells, either through Kir channels or through the Na^+^/K^+^/2Cl^−^ cotransporter, are not responsible for the light‐evoked ECS α decrease. Indeed, blocking Kir channels or the Na^+^/K^+^/2Cl^−^ cotransporter leads to an increase, not a decrease in the light‐evoked ECS α reduction. These results are in agreement with findings which demonstrate that neither Kir channels (Haj‐Yasein et al., 2011; Larsen et al., 2014) nor the Na^+^/K^+^/2Cl^−^ cotransporter (Larsen et al., 2014) are responsible for ECS α decreases in brain slices evoked by electrical stimulation of neurons. Nor do AQP4 water channels contribute to the light‐evoked α decrease. This finding is consistent with previous work demonstrating that activity‐dependent shrinkage of the ECS in the brain is not dependent on AQP4 (Haj‐Yasein et al., 2012; Toft‐Bertelsen et al., 2021).

Instead, our results indicate that the Na^+^/HCO_3_^−^ cotransporter is largely responsible for generating the ECS α decrease. Blocking the Na^+^/HCO_3_^−^ cotransporter by the removal of HCO_3_^−^ from the aCSF nearly abolishes the ECS α decrease, reducing the decrease by 95.6%. This effect cannot be attributed to a change in neuronal activity as removal of HCO_3_^−^ did not reduce light‐evoked RGC responses.

Inhibiting the monocarboxylate transporter also reduced the ECS α decrease, but only by 32.5%. This suggests that the monocarboxylate transporter may also contribute to the α decrease, as it does in the brain (Larsen & MacAulay, 2017). However, 4‐CIN, used to block the transporter, also decreased light‐evoked neuronal activity by ~15%. With this decrease in neuronal responsiveness, it is difficult to assess how much of the 4‐CIN‐induced reduction in the light‐evoked α decrease is due to direct block of a monocarboxylate transporter swelling mechanism and how much is due to the reduction of neuronal activity.

These results differ from findings in the brain, where both the Na^+^/HCO_3_^−^ cotransporter and the monocarboxylate transporter contribute to the generation of evoked ECS α decreases, but only partially (Larsen & MacAulay, 2017). Inhibiting the Na^+^/HCO_3_^−^ cotransporter reduced the evoked α decrease in the brain by ~25% while inhibiting the monocarboxylate transporter similarly reduced the α decrease by ~25%. Our results show that the action of the Na^+^/HCO_3_^−^ cotransporter can completely account for activity‐driven ECS α decreases in the retina.

Epileptiform activity in the brain is accompanied by a significant shrinkage of the ECS (Lux et al., 1986). Colbourn et al. (2021) have recently demonstrated that this shrinkage, as well as transient drops in ECS α superimposed on the sustained shrinkage (rapid volume pulsation) are generated by the Na^+^/HCO_3_^−^ cotransporter. Thus, the ECS shrinkage seen during epileptiform activity in the brain arises from the same mechanism that generates the light‐evoked ECS α decrease in the retina. Colbourn et al also demonstrated that Kir channels, the Na^+^/K^+^/2Cl^−^ cotransporter type 1, the K^+^/Cl^−^ cotransporter, and AQP4 channels do not contribute to the ECS shrinkage accompanying epileptiform activity.

The Na^+^/HCO_3_^−^ cotransporter transports either 2 or 3 HCO_3_^−^ along with 1 Na^+^ across the cell membrane (Newman & Astion, 1991; Romero & Boron, 1999; Theparambil et al., 2014). Thus, the cotransporter is electrogenic and its activity will be altered when a cell is depolarized. When neurons are stimulated they generate a K^+^ efflux which raises extracellular K^+^ levels. This depolarizes cells, particularly astrocytes and Müller cells, which are almost exclusively permeable to K^+^ (Newman, 1985). Glial cell depolarization will either increase the influx of HCO_3_^−^ and Na^+^ into the cells through the electrogenic Na^+^/HCO_3_^−^ cotransporter or reduce the efflux of HCO_3_^−^ and Na^+^, depending on the stoichiometry of the transporter and the Müller cell membrane potential. In either case, this change in cotransporter activity may lead to a change in water flux across the plasma membrane that would cause cell swelling.

This mechanism of Na^+^/HCO_3_^−^ cotransporter action may account for why the light‐evoked ECS α decrease actually becomes larger when Kir channels are blocked. Inhibition of Kir channels will depolarize Müller cells (Newman, 1989), leading to greater activation of the Na^+^/HCO_3_^−^ cotransporter and to increased cell swelling. It is less clear why light‐evoked ECS α decreases become larger when the Na^+^/K^+^/2Cl^−^ cotransporter is blocked.

The Na^+^/HCO_3_^−^ cotransporter is highly expressed in Müller cells but has not been detected in retinal neurons (Bok et al., 2001; Newman, 1996, 1999) and it is likely that Müller cells are responsible for generating the ECS α decrease in the retina. This was confirmed by monitoring light‐evoked Müller cell swelling. We found that light stimulation generated an increase in the volume of Müller cell processes in the IPL that had a similar time course and onset latency as the ECS α decrease.

A quantitative estimate of the contribution of Müller cells to the ECS α decrease can be made based on our results and previously published measurements of retinal tissue parameters. Within the middle portion of the IPL, Müller cells occupy ~10.5% of total tissue volume (Rasmussen, 1972) while ECS α equals ~12.2% (Kuo et al., 2020). These measurements include the stalk and fine processes of Müller cells but not cell somata, which are in the inner nuclear layer. Thus, a Müller cell volume increase of 6.3% would result in an ECS α decrease of 5.4%. This represents 62% of the ECS α decrease of 8.7% observed in our experiments. The discrepancy may indicate that the swelling of other cells besides Müller cells also contributes to the ECS α decrease. Alternately, the discrepancy may arise because our method of measuring Müller cell swelling in individual cells may underestimate the true extent of the response. For instance, our measurement of Müller cell swelling assumes that the concentration of dye in cell processes remains constant as the processes swell. A decrease in dye concentration accompanying process swelling, which is likely to occur, would result in an underestimation of the true magnitude of the swelling.

## 5 CONCLUSION

The light‐evoked ECS α decrease observed in the retina is generated, in large part, by the activation of the Na^+^/HCO_3_^−^ cotransporter in Müller cells, with a possible contribution of the monocarboxylate transporter. This mechanism differs from that observed in the brain, where several mechanisms, including the Na^+^/HCO_3_^−^ cotransporter, the monocarboxylate transporter, and other, unidentfied mechanisms all contribute to ECS α decreases evoked by neuronal activity (Larsen & MacAulay, 2017). Activity‐dependent, glial‐evoked ECS α decreases, both in the retina and brain, may contribute significantly to CNS function, modulating the diffusion of neurotransmitters and gliotransmitters by volume transmission and the activation of perisynaptic receptors by diffusion from the synaptic cleft.

## CONFLICT OF INTEREST

The authors declare no conflict of interest.

## Data Availability

The data that support the findings of this study are available from the corresponding author upon reasonable request.
